# Supplementary material for: Knowledge Beacons: Web services for data harvesting of distributed biomedical knowledge
Source: PLoS One. 2021 Mar 23;16(3):e0231916. doi: 10.1371/journal.pone.0231916 (PMC7987184; doi:10.1371/journal.pone.0231916)
Supplement: Attachment — Submitted filename: Response to Reviewers.pdf [file pone.0231916.s001.pdf]

PONE-D-20-09379: Knowledge Beacons: Web Service Workflow for FAIR Data Harvesting of Distributed Biomedical Knowledge

Response to Reviewers

Reviewer #1: 1- Knowledge Beacons is an API (REST web service) so calling it a workflow is a little bit stretched. I would remove the term workflow from the title and abstract. It can be one of steps in a bigger workflow but the article is about the API, not the workflow.
2- FAIR part of the API is partially fulfilled. It is available (findable, accessible in public repo, versioned), uses community standards (OpenAPI...) but you don't use or enforce agreed controlled vocabularies or ontologies in order to increase interoperability (reusable in FAIR) with external resources (in a bigger framework). Since you are mentioning FAIR directly in the title, more effort should be put in describing why and how this API and its (meta)data are FAIR.
3- I would like to see more API example calls (on some website or described in git repository) and a discussion (advantages/disadvantages) of using this API instead of calling directly Monarch db APIs (BioLink API).
4- Discussion lines 221-232 should be moved away from the end of the discussion since it deals with design choices of other projects and misses the connection with the proposed API
5- End of discussion (lines 237-245) talks about two other projects (reasoner API ad explorer beacon) but again it does not fit with the rest of the work (presentation of the knowledge beacons API)

Commented [RB1]: Done. Generally removed from paper.

Commented [RB2]: Stronger emphasis made of Biolink Model standard usage in the API.

Commented [RB3]: FAIR details added to Discussion section

Commented [RB4]: Github repository documentation enhanced.

Commented [RB5]: We have added some additional compare and contrast with the Monarch Biolink API.

Commented [RB6]: This section removed, although "Linked Open Fragments" mentioned/cited in the introduction.

Commented [RB7]: Removed

Reviewer #2: This paper describes the development of REST APIs to wrap 6 widely used knowledge sources (SemMedDB, Monarch DB, HMDb, Rhea DB, SMPDb and ndex) into knowledge beacons. This work serves as an initial first step towards the uniform integration of the resources mentioned herein in other software, which in turn will have a clear benefit towards the use of standardized vocabularies and relational structures in knowledge graphs - a common issue impeding the extensive usage for many of them in their current form - while serving the principles of FAIRness in data dissemination.

The manuscript does a very good work in describing the technical implementation of the beacons and also gives clear explanation of usage either with keywords or CURIE concepts. Results returned by a beacon follow the biolink model, which is a new and exciting effort towards standardazing and allowing the interoperability of knowledge graphs. The authors have also discussed any limitations of their method in the discussion section extensively.

Minor revisions
1. Even though the authors have not conducted an extensive assessment of the method's efficiency, it would be nice if they could provide 1-2 examples of query performance time for 2 different knowledge sources (since query times should be different for those). Perhaps a minimum and maximum recorded time on their setting would be a good indication (since indeed internet latency and bandwidth limitations can lead to big variations). It's undestandable that this implementation will not be as fast as some lower-level query interface, so providing some examples for query times will convince potential users that even though slower, its usage is not time-prohibitive and the benefits it provides are enough to overcome any issues of this type.

Commented [RB8]: Informal testing of query performance of all the reference beacons at https://kba.ncats.io/beacon/* generally exhibited a query performance of a fraction of a second, or at most, 1-2 seconds. We have deemphasized performance concerns, at least, for raw Beacons as hosted on adequate server infrastructure (as the reference beacons). The performance of our prototype Knowledge Beacon Aggregator (KBA) is slower primarily because of the significant extent of cross-dataset integration it performs. Client User Interfaces on top of the KBA add additional overheads to performance.

2. The authors mention in their paper that "conversion of Beacon statement results into RDF format is easily accomplished". Do the authors provide scripts for these conversions on their github page? If yes I think it should be referenced at that point, and if not they should point the

PONE-D-20-09379: Knowledge Beacons: Web Service Workflow for FAIR Data Harvesting of Distributed Biomedical Knowledge

reader to sourcecode that allows this conversion.

3. In the discussion section the authors refer to Linked Data Fragments, but I feel that this paragraph is lacking any connections to the rest of the manuscript and the project the way it is written. I would like to request from the authors to rewrite this paragraph so that the connection is clearer.

4. In the Results Section, subsection "Beacon implementations" numerous additional software are described in the first page, and the authors point only to the github pages of these. Are there any related publication where the authors could also point to for any of those? If yes, please add the references to those in that paragraph.

5. In the discussion section the authors mention the use of a "knowledge beacon aggregator". It would be nice if they could provide an example of how the aggregator can integrate the results from multiple beacons and what are the requirements for the results to be aggregated (or if this is described in detail in the github page of the tool, point to that description in that section).

I have two more suggestions rather than comments to the authors, which they can incorporate either in their manuscript or in their github page.

6. First, I would like to suggest to the authors to give a more concrete example of a biological application of their method, a use-case scenario where, for example, someone could use the API to extract knowledge and how that would look. Even though the technical implementation is very well described in the paper it would be nice if someone could see a more concrete example of the applicability of the knowledge beacon in real world scenarios. This can be easily incorporated into their already described sample workflow, where the two-step process is shown, but the usability of the results returned is not described in detail.

7. Following on the latter, and specifically for the ndex swagger, it would be nice to mention how this can be complementary to the core Cytoscape API, CyREST (Ono, Keiichiro, et al., 2015), since ndex goes hand in hand with Cytoscape.

Commented [RB9]: No such scripts are, as yet, coded thus we removed this sentence (and the conjecture) from the paper.

Commented [RB10]: Removed from manuscript.

Commented [RB11]: No other papers have been submitted/published for beacon implementations

Commented [RB12]: The Github repository documentation for the Knowledge Beacon Aggregator was enhanced.

Commented [RB13]: New example given in the Results section for another human disease, PSP.

Commented [RB14]: Thanks for the suggestion. We have added a few references to Cytoscape and CyREST in the paper. This is an interesting idea. Given that the "knowledge" being retrieved has an inherent (sensa computer science) graph nature - concept nodes and relationship edges – one could contemplate use of a Cytoscape view of the data. That said, we don't currently have any beacon data output conversion script to cast it into a form directly consumable and viewable in Cytoscape. The availability of an nDEX Beacon wrapper does, however, permits interoperable software integration between ndex hosted data and other available knowledge sources. CyREST, however, seems specific to Cytoscape accessible data sources. Maybe adding Knowledge Beacons as a Cytoscape plug-in graph source, or conversely, wrapping CyREST as a Beacon, could bridge this gap.
